# Ameliorative effects of *Portulaca oleracea* L. (purslane) and its active constituents on nervous system disorders: A review

**DOI:** 10.22038/IJBMS.2022.65764.14464

**Published:** 2023-01

**Authors:** Jalileh Jalali, Mahboobeh Ghasemzadeh Rahbardar

**Affiliations:** 1 Department of Education District 4, Mashhad, Iran; 2 Pharmaceutical Research Center, Pharmaceutical Technology Institute, Mashhad University of Medical Sciences, Mashhad, Iran

**Keywords:** Alzheimer’s disease, Depression, Epilepsy, Herbal medicine, Hypoxia, Pain, Parkinson’s disease

## Abstract

Nowadays, the global interest in the use of herbal medicines and their main components in developing novel effective medications with fewer adverse effects is rising. Precise medicinal plants have potential advantageous applications for several neurodegenerative disorders. *Portulaca oleracea* L. (purslane) belongs to the *Portulacaceae Juss* family. In folk medicine, it has been used as a febrifuge, antiseptic, vermifuge, and in treating arthritis, burns, cough, headache, intestine, stomach, liver disorders, as well as shortness of breath. Pharmacological investigations have also disclosed its antioxidant, anti-inflammatory, anti-apoptotic, immunomodulatory, antidepressant, anxiolytic, and neuroprotective properties. The current work prepared an updated and broad literature review on purslane highlighting its therapeutic effects on some nervous system disorders. It has been shown that *P. oleracea* and its active constituents have considerable neuroregenerative, neuroprotective, and antinociceptive properties. Accordingly, our team classified and discussed the outcomes of some nervous system disorders comprising Alzheimer’s disease, Parkinson’s disease, depression, epilepsy, anxiety, psychosis, drug dependence, hypoxia, and pain; although, additional preclinical and clinical assessments are necessary to reinforce the beneficial effects of purslane on nervous system disorders.

## Introduction

Nervous system disorders are abnormalities in the peripheral or central nervous system’s structure or function (1). Trauma, metabolic malfunction, infection, or inherited factors may all play a role in these disorders. Many scientific investigations and discoveries are aimed at reducing the severity and frequency of neurological illnesses, mental illness, and substance abuse. In ancient remedies, herbal medications and natural items were utilized (2). Because herbs have fewer side effects and complications, researchers have focused more on them in medicine development in recent decades (3). Pharmacological and therapeutic research projects have been developing worldwide in response to rising demand (4-6).

**Figure 1:**
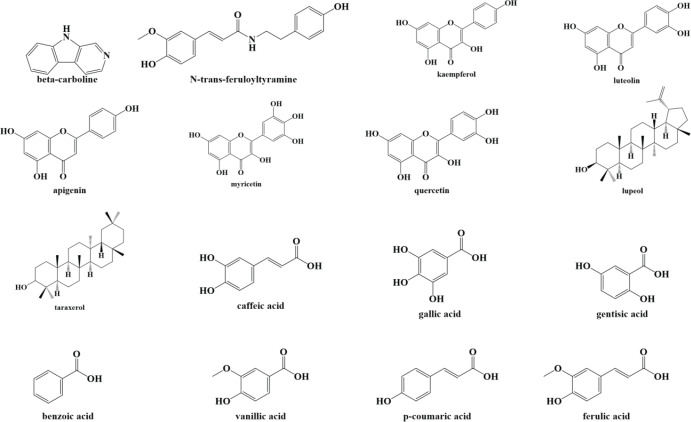
Chemical structure of some of the main constituents of *Portulaca oleracea* L

Purslane (*Portulaca oleracea* L.) is a member of the *Portulacaceae Juss* family. It is found all across the world and grows primarily in the tropics and subtropics, with its origins in Africa and South America (7, 8). The term “Portulaca” comes from two Latin words ‘Porto’ which means “to carry” and “lac” which means “milk”, and it refers to the existence of milky juice in this herb (8, 9). According to several studies purslane is popular for its medicinal (10, 11), nutritional (12, 13), and phytoremediation (14, 15) effects. As stated by phytochemical studies this herb is one of the richest terrestrial sources of β-carotene, glutathione (GSH), ω-3, ω-6 fatty acids, tocopherols, and ascorbic acid (8, 16), indicating its nutraceutical potential. Betalains, terpenoids (portuloside A, portulene, lupeol, friedelane, taraxerol), lignans, flavonoids (apigenin, genistein, genistin, kaempferol, luteolin, myricetin, quercetin), anthocyanins (delphinidin-3,5-glucoside, cyanidin-3,5-glucoside, pelargonidin-3,5-glucoside, delphinidin-3-glucoside, cyanidin-3-glucoside, pelargonidin-3-glucoside), phenolic acids (caffeic acid, p-coumaric acid, ferulic acid, gallic acid, gentisic acid, benzoic acid, anisic acid, vanillic acid), catecholamines, and alkaloids (oleraceins A, B, C, D, E, oleracimine, oleracimine A, oleracone A, oleracone B, β-carboline, N-trans-feruloyltyramine, dopamine, dopa, noradrenaline) are only a few of the specialized metabolites found in purslane (17-19) (Figure 1). Some of these metabolites have been demonstrated to possess health-promoting benefits for humans (20-24). It was reported that *P. oleracea* has been utilized in traditional cuisine and folk medicine as a vermifuge, antiseptic, febrifuge, also in managing headache, cough, burns, arthritis, shortness of breath, as well as intestine, stomach, and liver disorders in numerous parts of the world since ancient times (9). Several ethnobotanical studies suggest that it is used as an important medicine by indigenous communities to treat a variety of ailments, including cardiovascular and kidney diseases, headaches, diabetes, ulcers, urinary infections, diarrhea, as well as insect and snake bites (17, 25, 26). Moreover, its usage as an ethnomedicinal plant has been reported from practically every continent, implying that it is extremely important in native people’s healthcare (8). A great number of studies, either on animal models or on cultured cells, show that purslane and its main constituents have a wide spectrum of therapeutic characteristics such as antioxidant (27-29), anti-inflammatory (30, 31), immunomodulatory (32), antidiabetic (33), antimicrobial (34), anticancer (35), neuroprotective (36-40), antidepressant (41), and anxiolytic (42). 

Concerning the safety of *P. oleracea*, it is important to note that the median lethal dose (LD_50_) value of the aqueous extract of purslane leaves prescribed through a stomach tube to adult male Sprague Dawley rats was 4500 mg/kg of their body weight (43). Furthermore, the LD_50_ value of ethanolic extract of the whole plant was 500 mg/kg in Swiss Albino mice, and in an acute toxicity test on mice, the LD_50_ value of methanolic extract was 1853 mg/kg (44).

This review summarizes the multiple pharmacological effects of purslane and its principal component in the treatment and management of nervous system disorders to bring scientists’ attention to focus and link the value of *P. oleracea* L. from fundamental sciences to patients’ beds.

***Methods***

Several databases were used in this study, including Scopus, Google Scholar, and PubMed. All of the articles included (*in vitro*, *in vivo*, and clinical trials) were collected from the time of inception until March 2022. The search terms included “*Portulaca oleracea* L.”, “purslane”, “benzoic acid”, “oleraisoindole”, “oleraciamide”, “betacyanins”, “oleracein E”, “nervous system”, “depression”, “memory”, “Alzheimer’s disease”, “anxiety”, “psychosis”, “drug dependence”, “epilepsy”, “seizure”, “anticonvulsant”, “addiction”, “pain”, “neuropathic pain”, “antinociceptive”, and “analgesic”.

***Therapeutic effects of purslane on nervous system disorders***

*Alzheimer’s disease and memory impairments*

Alzheimer’s disease is a long-term neurological disorder that accounts for the majority of dementia cases. Short-term memory loss, confusion, loss of motivation, mood changes, and behavioral difficulties are all common symptoms of Alzheimer’s disease (45). As their condition worsens, those with Alzheimer’s disease frequently retreat from their families and society. The underlying processes of Alzheimer’s disease are unknown, and most present pharmacological therapy is based on the cholinergic theory. According to the cholinergic hypothesis, Alzheimer’s disease is caused by a decrease in the synthesis of the neurotransmitter acetylcholine (ACh) (46). Inhibiting the enzyme acetylcholinesterase (AChE), which breaks down ACh, is a viable technique for treating people with Alzheimer’s disease. Alternative explanations exist, such as the amyloid and tau hypothesis, although AChE is a popular enzyme target among scientists (47). Other possible underlying mechanisms of memory deficit are reported to be oxidative stress and inflammation (48, 49).

In a microplate assay, two new natural compounds, benzoic acid, 4-[[(2-hydroxyethyl)amino] carbonyl]-methyl ester and benzoic acid, 3-[[(2-hydroxyethyl)amino]carbonyl]-methyl ester were isolated from purslane with antioxidant and anticholinesterase properties (50). Moreover, it was observed that oleraisoindole A, a trace alkaloid, from *P. oleracea* L. has dose-dependent anticholinesterase activity (51). Similarly, another study discovered two new purslane amide alkaloids, oleraciamide G and oleraindole D, with dose-dependent anticholinesterase activity (52). In addition, two new esters, ethyl(7E,9E)-6-oxooctadeca-7,9-dienoate and 1-ethyl 7-(4-octyl-5-oxocyclopenta-1,3-dien-1-yl) heptanedioate, with dose-dependent anticholinesterase effects, were discovered (53). 

The neuroprotective effect of polysaccharides extracted from purslane was investigated against neurotoxicity *in vitro* (PC12 cells) and *in vivo* (rats). Treating PC12 cells with polysaccharides extracted from purslane elevated the survival of these cells and attenuated reactive oxygen species (ROS) generation. Furthermore, polysaccharides extracted from purslane were shown to improve lead-induced learning and memory impairments by increasing platform crossing times, decreasing escape latency, and decreasing dendritic spine loss (54) (Table 1).

**Figure 2:**
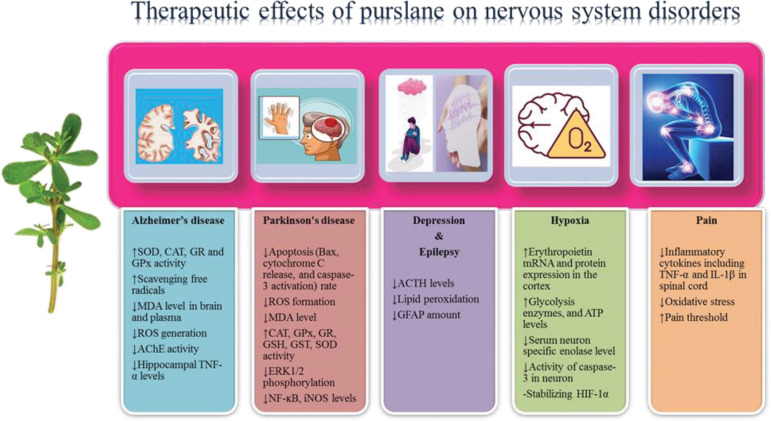
Therapeutic effects of *Portulaca oleracea* L. on nervous system disorders

It has been reported that the prescription of betacyanins derived from purslane to mice with learning and memory impairment lowered cognitive impairment by increasing superoxide dismutase (SOD), catalase (CAT), glutathione reductase (GR), and glutathione peroxidase (GPx) amounts in mice brain (55). In another similar research, it was indicated that receiving betacyanins derived from purslane in mice with cognition deficits resulted in augmented levels of SOD, CAT, GR, and GPx, as well as reduced malondialdehyde (MDA) levels in their brain tissues. Betacyanins also significantly attenuated learning and memory impairments. The authors stated that the effect of betacyanins on ameliorating cognition deficits in mice was greater than that of vitamin C (56) (Figure 2). It has also been stated that the prescription of ethanolic extract of *P. oleracea* to rats improved spatial memory and learning ability and attenuated oxidative stress in neurons (57). Besides, it has been found that purslane seeds inhibited the progression of brain damage (including enlarged vacuolar spaces of neuropil, extended perivascular space, congestion and dilation of blood vessels, loss of neurons, and structural changes in the frontal cortex) induced by hyperlipidemia in the rat by scavenging free radicals (58). The results of a study illustrated that prescription of aqueous extract of *P. oleracea* L. to diabetic ovariectomized rats enhanced their spatial cognitive performance, total distance traveled at the probe, and decreased neurobehavioral dysfunction, anxiety, and non-functional masticatory activity (59). It has also been claimed that prescription of hydro-alcoholic extract of purslane to Wistar rats amended passive avoidance learning and memory, besides declining hippocampal tumor necrosis factor-α (TNF-α) levels (60). It was observed that prescription of phenolic extract of purslane containing indoline amides to mice with a cognitive deficit caused a significant increase in lifespan, spatial memory, learning ability, and CAT level in the brain and plasma. It also decreased MDA levels in the brain and plasma, as well as hippocampal morphological damage. However, it had no effect on AChE activity in the brain (61). Administration of purslane to diabetic rats along with exercise training amended neurobehavioral deficits by increasing exploration and reducing passive avoidance memory deficit and anxiety (62). This group of researchers conducted another study and evaluated the effect of co-administration of purslane and *Plantago psyllium* along with submaximal swimming training on memory deficit in diabetic rats. The obtained results showed that swimming training could not ameliorate learning and memory indices, whereas co-administration of *P. oleracea* and *P. psyllium* with swimming training amended diabetic rats’ exploratory behavior, general locomotor activity, and passive avoidance memory (63). A recent study reported that prescription of aqueous extract of purslane to rats with memory decline caused a significant increase in cognitive memory, the number of intact neurons in the brain, as well as SOD and CAT levels. It also considerably decreased brain histopathological injury, MDA amount, TNF-α, and nuclear factor kappa B (NF-ĸB) concentrations (64). 

These results indicate that different extracts of *P. oleracea* and its compounds might be promising agents in treating and managing cognitive impairments and aging through different mechanisms including antioxidant (scavenging free radicals, decreasing ROS generation and MDA amount as well as increasing SOD, CAT, GR, and GPx levels in the brain), anti-inflammatory (attenuating hippocampal TNF-α level), and anticholinesterase (reducing AChE activity in the brain) effects. However, a lack of clinical trials in this field is a significant limitation, and more clinical studies are needed to confirm the beneficial effects of purslane in human cognitive impairments.

***Parkinson’s disease***

Parkinson’s disease is the 2^nd^ most common neurodegenerative ailment, affecting about 3% of the population over 65 years old. Parkinsonism is a clinical syndrome characterized by frozen phenomena, flexed posture, loss of postural reflexes, muscle rigidity, resting tremor, and bradykinesia. Patients with Parkinson’s disease might also suffer from non-motor symptoms including depression, fatigue, sleep difficulties, anxiety, and dementia. The loss of neuromelanin-containing dopamine neurons in the substantia nigra and formation of Lewy bodies in the cytoplasm of cells are neuropathological markers of Parkinson’s disease (65, 66). The substantia nigra pars compacta provides the densest dopaminergic innervation to the striatum, the major input nucleus of the basal ganglia. In Parkinson’s disease, the loss of dopaminergic innervation impairs the capacity of the two main striatal projection systems to respond adequately to cortical and thalamic impulses, resulting in hypokinetic symptoms (67). When dopamine or glutamate receptors are stimulated in striatal neurons, the Ras/extracellular signal-regulated protein kinase 1/2 (ERK 1/2) signaling cascade is triggered, which has been linked to the development of dyskinesia (68). The development of Parkinson’s disease appears to be influenced by oxidative stress, inflammatory factors, and apoptotic pathways, besides the accumulation of α-synuclein and aquaporin 4. Environmental and genetic variables, can both play a role in illness progression. Some of the most well-known environmental toxins that cause Parkinson’s disease include heavy metals, herbicides, and pesticides (66, 69).

The effect of oleracein E which was derived from purslane was assessed on rotenone-induced Parkinson’s disease through *in vitro* and *in vivo* models. The results of *in vitro* study disclosed that oleracein E decreased lactate dehydrogenase (LDH) release [LDH is commonly employed in cytotoxicity experiments and is thought to be a key biomarker of cell membrane integrity (70)] and apoptosis by reducing Bcl-2-associated X (Bax) amount, caspase-3 activation, and cytochrome C release, ROS, and ERK1/2 phosphorylation in SH-SY5Y cells. *In vitro* part of the study indicated that oleracein E improved motor function, SOD activity, tyrosine hydroxylase (TH)-positive neurons, the density of dopaminergic fibers in the substantia nigra pars compacta and reduced MDA content, ERK1/2 phosphorylation in the midbrain as well as striatum in mice (36) (Table 2).

Investigating the anti-Parkinson’s disease effect of purslane on rotenone-induced neurotoxicity in striatum revealed that this herb could significantly increase striatum level of Na^+^/K^+^-adenosine triphosphate (ATP)ase activity, CAT, GPx, GR, GSH, glutathione-S-transferase (GST), SOD, and lowered protein carbonyl and hydrogen peroxide (H_2_O_2_) (71). This group of researchers conducted another similar study and they reported that purslane remarkably decreased apoptosis by increasing B-cell lymphoma-2 (Bcl-2) and lowering caspase-3 in this part of the brain. It also reduced inflammation via inhibiting inducible nitric oxide synthase (iNOS) and NF-ĸB expression and oxidative stress through attenuating ROS, nitrite/nitrate, and lactate dehydrogenase, as well as thiobarbituric acid reactive substances (72). The effect of aqueous and ethanolic extracts of *P. oleracea *was assessed on lesions of dopaminergic neurons in rats. The data illustrated that both extracts could improve motor recovery and attenuate TH-cell loss, but these properties were more apparent for the aqueous extract (38). In an *in vivo* study, dUCH-knockdown Drosophila was used to assess the capacity of aqueous extract of *P. oleracea* for Parkinson’s disease treatment. The obtained data revealed that purslane could considerably improve locomotor ability in the larval stage and reduce dopaminergic neuron degeneration and disease progression in the adult stage (44). Assessing the anti-Parkinson effect of purslane seed methanolic extract in rodents showed that the extract could significantly scavenge free radicals such as hydroxyl anions and reduce ROS formation, lipid peroxidation, as well as cataleptic behavior (73).

Overall, these findings highlight the pro-survival effect of purslane, its extracts, and oleracein E in the midbrain and striatum, as well as its potential as a prophylactic against brain damage and neurodegenerative illnesses linked to oxidative stress. The anti-Parkinson effect of this herb is suggested to be carried out by decreasing oxidative stress (increasing CAT, GPx, GR, GSH, GST, SOD, scavenging free radicals including hydroxyl anions, decreasing ROS, MDA, protein carbonyl, H_2_O_2_, and lipid peroxidation), inflammation (attenuating NF-ĸB and iNOS), apoptosis (reducing Bax, cytochrome C release, and caspase-3 activation, and boosting Bcl-2 amount), balancing cell signaling pathways, LDH release, increasing density of dopaminergic fibers in the substantia nigra pars compacta, motor function, TH-positive neurons, and striatum level of Na+/K^+^-ATPase activity. Though the number of studies especially the clinical trials is limited, more investigations are necessary to validate the advantageous effects of purslane in managing Parkinson’s disease.

***Depression***

Depression is now proposed to be the second most common disorder after cardiovascular disease, causing significant socioeconomic distress. Several overlying physiological interconnections are responsible for the pathophysiology of depression (74). In earlier decades, the most common theories on the pathophysiology of depression focused on monoamine expression and receptor dysfunction, decreased monoamine synthesis, or failures of secondary messenger systems such as G proteins or cyclic adenosine monophosphate (cAMP). Cortisol augmentation and its detrimental effects on neurogenesis by lowering endogenous opioid function, decreasing brain-derived neurotropic factor (BDNF), changing gamma-aminobutyric acid (GABA)ergic or/and glutamatergic transmission, irregular circadian rhythm, and cytokine or steroidal changes have all received more attention in recent years (75, 76). Moreover, some evidence has disclosed the contributions of the corticotropin-releasing hormone, adrenocorticotropic hormone (ACTH), and cortisol disturbances in the induction of postpartum depression (77).

Because purslane is high in minerals including lithium, folate, calcium, potassium, and magnesium, this plant is supposed to possess antidepressant properties (78). On a dry weight basis, the plant contains up to 16% antidepressant ingredients (79). In an *in vivo* study, it was observed that prescription of aqueous purslane extracts significantly shortened the immobility times in the forced swim test and tail suspension test. But, pre-treatment with NBQX (AMPA antagonist) considerably reversed the effect of purslane extract (41). In another study forced swim test was employed to assess the antidepressant effect of *P. oleracea* extract in rats, followed by an examination of the ACTH amount. The extract effect was comparable with diazepam, which reduced immobility time and ACTH levels (80) (Table 3).

As a result, it is possible to propose that *P. oleracea* exerts its anti-depressive effect by lowering ACTH levels. However, research in this field is limited, and more research is needed to confirm its anti-depressive properties and underlying mechanisms.

***Anxiety***

A state of inappropriate or excessive worry that is frequently accompanied by agitation, distraction, tension, irritation, and sleep difficulties is known as anxiety. By over-activating the hypothalamic-pituitary-adrenal axis and the autonomic nervous system, this excessive reaction to environmental cues can cause bodily symptoms of anxiety such as sweating, shortness of breath, raised blood pressure, a rapid heartbeat, and nausea. The basis of medication therapy for anxiety disorders continues to be benzodiazepines. But they also have significant negative effects, including pharmacological dependence, amnesia, ataxia, and drowsiness (81).

It was observed that when mice were given an ethanol extract of purslane, the percentage of time spent and arm entries into the open arms of the elevated plus maze were much higher than those of the controls. In addition, none of the groups’ myorelaxant effects or locomotor activity differed from those of the saline controls. Flumazenil, a GABAA antagonist, was also able to counteract the anxiolytic-like effects of the purslane extract but not WAY 100635, a 5-HT1A receptor antagonist (81). Moreover, it was shown that intraperitoneal injection of aqueous extract of purslane to mice could reduce anxiety reaction and increase the number of entrances and percent of time spent in the open arm in comparison with the control group dose-dependently (42) (Table 3).

These findings indicate that purslane is a potent anxiolytic agent, and it might be suggested that purslane exerts its anxiolytic effect by affecting the GABAergic system.

***Epilepsy***

Epilepsy is defined as a group of seizures that occur spontaneously and repeatedly as a result of abnormal synchronic and neuronal function in the brain (3). It is one of the most frequent central nervous system (CNS) disorders, affecting about 1% of the global population (82). The development of epilepsy in patients and animal models is linked to an imbalance between inhibitory and excitatory neurotransmission in the brain, which could be caused by an upsurge in glutamatergic and/or a decline in GABAergic transmission (83). Glial fibrillary acidic protein (GFAP) is a cytoskeletal protein that is abundantly expressed in astroglial cells and neural progenitor cells. Glial cells have a role in epileptogenesis by releasing inflammatory proteins such as interleukins and chemokines, which can promote hyper-excitability. Any alteration in the correct astrocyte makes these newly born neurons vulnerable to aberrant connections, which can lead to hyper-excitability (84). The existing anticonvulsants have some problems, including teratogenicity and other dose-related side effects. The ancient medical system offers a wide range of solutions for these issues, as well as a great supply of medicinal plants that have fewer side effects, which are gaining favor all over the world (85). Furthermore, the previous investigations illustrated the effects of different herbs or their active components in managing epilepsy (86, 87). 

In a maximum electroshock model in mice, an aqueous extract of *P. oleracea* leaves reduced the recovery time and the duration of the hind limb tonic extension phase. It also sped up the start and duration of pentylenetetrazol-induced clonic convulsions (82). The administration of aqueous extract of purslane seeds to epileptic rats resulted in decreased epileptic hyper-excitability, GFAP, and lipid peroxidation (84) (Table 3).

Therefore, it could be concluded that *P. oleracea *and its seeds reveal their anticonvulsant effect by reducing oxidative stress, expression of GFAP, astroglial cell function, and epileptic hyper-excitability. However, more studies are required to confirm the anti-epileptic property of this herb and the underlying mechanisms.

***Hypoxia***

Hypoxia is a condition in which oxygen levels in body tissues are low, and it has been linked to the pathophysiology of acute cardiovascular disease, stroke, and mountain sickness, which are among the top reasons for death worldwide (88). The mammalian brain is extremely vulnerable to hypoxia-induced neuronal injury. The death of neurons as a result of hypoxia can cause a range of neurological problems. As a result, anti-hypoxic medications have become the focus of an increasing number of investigations. Detecting innovative cellular pathways stimulated in hypoxia neuronal settings will shed new light on hypoxia neurological illness therapy options (89).

Erythropoietin promotes the survival, proliferation, and differentiation of erythroid progenitor cells, which in turn stimulate erythroid cell production (90). Other cell types, such as endothelial and neuronal cells, have been identified to express the erythropoietin receptor and contribute to the erythropoietin response (91). It has been well established that astrocytes and neurons make erythropoietin in the brain, and that endogenous erythropoietin in the brain has neuroprotective properties (92, 93). Erythropoietin has recently been proposed for clinical treatment of many ischemic–hypoxic cardiovascular and neurovascular diseases, though some studies have found that it may have a negative effect on patients with acute stroke (94-96). All of this suggests that modulating erythropoietin expression could be a potential way to treat ischemia–hypoxia neurovascular diseases.

Investigating the effect of purslane extracts on hypoxic nerve tissue unveiled that the extracts could significantly boost erythropoietin messenger ribonucleic acid (mRNA) and protein expression in the cortex, activities of pyruvate kinase, phosphofructokinase [two enzymes that control the glycolysis process], and LDH levels (78). It has been disclosed that administration of ethanolic extract of purslane to mice could dose-dependently increase the survival time in chemical and normobaric hypoxia models, glycolysis enzymes, and adenosine triphosphate (ATP). The purslane extract did not affect the pentobarbital sodium-induced sleeping time or motor performance, representing that the anti-hypoxic activity was unlikely to be attributed to sedation or motor abnormalities (25). This group of researchers also reported that administration of ethanolic extract of purslane to mice with hypoxia-induced neuro damage stabilized hypoxia-inducible factor-1α (HIF-1α), augmented neuron viability, expression of endogenous erythropoietin at both mRNA and protein levels, reduced caspase-3 activity in neurons, serum neuron-specific enolase [a catalytic enzyme located primarily in the cytoplasm of cells that is required for fermentation, glucose catabolism, and synthesis] level, as well as pathological damages (89). It has been reported that oral prescription of purslane seed oil to rats could pointedly decrease the damage of body tissue triggered by brain stroke and resulted in neurological protection (97)(Table 3).

To conclude, purslane extracts can increase glycolysis, erythropoietin expression, and reduce fermentation in nerve tissue/cells to protect them from hypoxic damage.

***Pain***

The ability of neural systems to point to threatening or existing tissue injury evolved into the neurophysiological process that generates nociceptive pain. Its supporting task necessitates immediate responsiveness and attention, which is achieved by inducing the withdrawal reflex, an innately unpleasant sensation, as well as emotional distress (98). Neuropathic pain is a type of pain that is produced by somatosensory system injuries or illnesses. It involves the activation of nociceptive pathways as well as induction of allodynia and hyperalgesia (99). According to some studies, oxidative stress (100), inflammation (101), and apoptosis (102) are the key underlying processes of pain.

It was reported that administration of hydro-alcoholic extract of purslane seeds increased pain threshold and reduced pain in tail-flick test in mice (103). An investigation compared the anti-inflammatory and antinociceptive properties of purslane leaf and seeds. The obtained data revealed that seed extract is more effective than leaf extract at reducing inflammation. In terms of analgesic efficacy, the leaf extract is more effective than seed extract in the early phase, whereas seed extract is superior analgesia in the late phase (104). Administration of purslane hydro-alcoholic extract to rats with neuropathic pain has been observed to attenuate pain-related behaviors, oxidative damage (MDA amount), and inflammatory cytokines including TNF-α and IL-1β in the spinal cord (105) (Figure 2).

Assessing the effect of purslane cream on nursing mothers revealed that this herb could significantly reduce nipple soreness and increase breastfeeding time in comparison with the lanolin group (106). Another clinical trial indicated that prescription of purslane ointment to elderly patients with chronic musculoskeletal pain resulted in the decreased severity of pain, besides reduced sensory and emotional dimensions of pain (107) (Table 4).

Subsequently, purslane might be an advantageous candidate for ameliorating pain by reducing oxidative stress and inflammation, even though more clinical evaluations are needed to elucidate its therapeutic effectiveness in humans.

**Table 1:** Effect of *Purtulaca oleracea* L. on Alzheimer’s disease and memory impairments

**Compound **	**Study design**	**Doses/Duration**	**Results**	**References**
benzoic acid, 4-[[(2-hydroxyethyl)amino] carbonyl]-methyl ester (1) and benzoic acid, 3-[[(2-hydroxyethyl)amino]carbonyl]-methyl ester isolated from * P. oleracea* L.	Microplate assay	20 to 60 µM	**↑**Antioxidant and anticholinesterase effects	(50)
Oleraisoindole A from *P. oleracea* L.	Microplate assay	2.5 to 40 µM	**↓** AChE activity	(51)
Oleraciamide G and oleraindole D from *P. oleracea* L.	Microplate assay	50 to 70 µM	**↓** AChE activity	(52)
ethyl(7E,9E)-6-oxooctadeca-7,9-dienoate and 1-ethyl 7-(4-octyl-5-oxocyclopenta-1,3-dien-1-yl) heptanedioate derived from *P. oleracea *L*.*	Microplate assay	60 to 70 µM	**↑** Anticholinesterase effects	(53)
Polysaccharides extracted from* P. oleracea *L*.*	*In vitro*, PC12 cells *in vivo*, Sprague-Dawley rats	50-400 μg/ml, 24 h 200, 400, 600 mg/kg, 30 days, p.o.	↑ Survival of PC12 cells, platform crossing times ↓ ROS generation, escape latency, loss of dendritic spine	(54)
Betacyanins from* P. oleracea* L*.*	*In vivo*, male Kunming strain mice	50, 100 mg/kg/day, 2 weeks, p.o.	↑ SOD, CAT, GR, and GPx amount in mice brain ↓ Cognitive impairment	(55)
Betacyanins from* P. oleracea* L*.*	*In vivo*, male Kunming strain mice	50, 100 mg/kg/day, 2 weeks, p.o.	↑ SOD, CAT, GR, and GPx ↓ Lipid peroxidation (MDA), learning and memory impairments	(56)
*P. oleracea* L. ethanolic extract	*In vivo*, Wistar rats	100, 200, 400, and 800 mg/kg, 14 days, p.o.	↑ Spatial memory and the learning ability ↓ Oxidative stress in neurons	(57)
Purslane seeds	*In vivo*, hyperlipidemic male albino rats	purslane seed 20% (20 g/100 ml water), 3 months, p.o.	↑ Scavenging free radicals ↓ Structural changes in the frontal cortex, loss of neurons, dilation and congestion of blood vessels, expanded perivascular space, and increased vacuolar spaces of neuropil	(58)
Aqueous extract of *P. oleracea* L.	*In vivo*, diabetic ovariectomized Wistar rats	300 mg/kg, 35 days, p.o.	↑ Spatial cognitive performance, total distance traveled at the probe ↓ Neurobehavioral dysfunction, anxiety, non-functional masticatory activity	(59)
Hydro-alcoholic extract of * P. oleracea *L*.*	*In vivo*, male Wistar rats	400 mg/kg, 14 days, p.o.	↑ Passive avoidance learning and memory ↓ Hippocampal TNF-α levels	(60)
Phenolic extract containing indoline amides from *P.* *oleracea* L.	*In vivo, * Kunming mice	360, 720 mg/kg, 8 weeks, p.o.	↑ Lifespan, spatial memory and learning ability, CAT level in brain and plasma ↓ MDA level in brain and plasma, hippocampal morphological damage	(61)
*P. oleracea *L*.*	*In vivo*, diabetic male Wistar rats	5% of diet, 12 weeks, p.o.	↑ Exploration ↓ Passive avoidance memory deficit and anxiety	(62)
*P. oleracea *L*.*	*In vivo*, diabetic male Wistar rats	5% of diet, 12 weeks, p.o.	↑ Exploration, general locomotor activity ↓ Passive avoidance memory deficit	(63)

AChE: acetylcholinesterase; CAT: catalase; GPx: glutathione peroxidase; GR: Glutathione reductase; MDA: malondialdehyde, PO: oral; ROS: reactive oxygen species; SOD: superoxide dismutase; TNF-α: tumor necrosis factor-alpha

**Table 2:** Effect of *Purtulaca oleracea* L. on Parkinson’s disease

**Compound **	**Study design**	**Doses/Duration**	**Results**	**References**
Oleracein E	*In vitro, *SH-SY5Y human neuroblastoma cells *In vivo, *mice	10 μM, 2 hr 15 mg/kg/d, 56 days, p.o.	↓ LDH release and the apoptosis (Bax, cytochrome C release, and caspase-3 activation) rate, ROS, ERK1/2 phosphorylation in SH-SY5Y cells ↑ Motor function, SOD activity, TH-positive neurons, and density of dopaminergic fibers in the substantia nigra pars compacta ↓ MDA content, ERK1/2 phosphorylation in the midbrain and striatum	(36)
Purslane aqueous juice	*In vivo, * male Wister albino rats	1.5 ml/kg, 12 days, p.o.	↑ Striatum level of Na^+^/K^+^-ATPase activity, CAT, GPx, GR, GSH, GST, SOD ↓ protein carbonyl and H_2_O_2_	(71)
Purslane aqueous juice	*In vivo, * male Wister albino rats	1.5 ml/kg, 12 days, p.o.	**↑** Bcl-2 **↓ **Caspase-3, NF-ĸB, iNOS, ROS, nitrite/nitrate and lactate dehydrogenase, thiobarbituric acid reactive substances	(72)
Aqueous and ethanolic extracts of *P. oleracea* L.	*In vivo, * male Wistar rats	200 and 400 mg/kg, 2 weeks, p.o.	**↑ **Motor recovery ↓ TH-cell loss	(38)
Aqueous extract of *P. oleracea * L.	*In vivo, *dUCH-knockdown Drosophila	25, 50, 100, and 200 μg/ml, 9 days	**↑ **Locomotor ability in the larval stage **↓ **Disease progression in the adult stage, dopaminergic neuron degeneration	(44)
*P. oleracea * L. seed methanolic extract	*In vivo, * Wistar rats and Swiss albino mice	200 and 400 mg/kg, 21 days, p.o.	↑ Scavenge free radicals including hydroxyl anions ↓ ROS, lipid peroxidation, cataleptic behavior	(73)

ATP: adenosine triphosphate; Bax: Bcl-2 associated X; Bcl-2: B-cell lymphoma-2; CAT: catalase; ERK1/2: extracellular signal-regulated kinase 1/2; GPx: glutathione peroxidase; GR: glutathione reductase; GSH: glutathione; GST: glutathione-S-transferase; H2O2: hydrogen peroxide; inducible nitric oxide synthase; LDH: lactate dehydrogenase; MDA: malondialdehyde; NF-ĸB: nuclear factor-kappa B, PO: oral, ROS: reactive oxygen species; SOD: superoxide dismutase; TH: tyrosine hydroxylase

**Table 3:** Effect of *Purtulaca oleracea* L. on depression, epilepsy, anxiety, psychosis, drug dependence, and hypoxia

**Compound **	**Study design**	**Doses/Duration**	**Results**	**References**
**Depression**				
Aqueous extract of *P. oleracea * L.	*In vivo, * male C57BL/6J mice	100, 500, or 1000 mg/kg, p.o.	**↓ ** Immobility times in forced swim test and tail suspension test	(41)
*P. oleracea* L. extract	*In vivo, * female Wistar rats	200 and 400 mg/kg, p.o.	**↓** Immobility time and ACTH levels	(80)
**Anxiety**				
Ethanolic extract of* P. oleracea *L.	*In vivo*, ICR mice	50, 100, 200 or 400 mg/kg, p.o.	**↑** Percentage of time spent and arm entries into the open arms of the elevated plus maze	(81)
Aqueous decoction extracts of * P. oleracea *L.	*In vivo*, mice	25, 50, and 75 mg/kg, IP	↑ Number of entrances and spent more percent time in open arm ↓ Anxiety reaction	(42)
Psychosis				
*P. oleracea *L.	Clinical trial, schizophrenic patients	1 g (capsule), daily, 8 weeks	- Improvement of psychological condition ↓ MDA levels	(108)
Drug dependence				
Aqueous extracts of * P. oleracea *L.	*In vivo*, albino mice	25, 50, 75 mg/kg, IP	↓ The number of jumping, weight loss	(109)
Epilepsy				
Aqueous extract of *P. oleracea* L. leaves	*In vivo, * albino mice	200, 400, and 600 mg/kg, p.o.	↓ Duration of recovery time and the hind limb tonic extension phase, onset and duration of clonic convulsion	(82)
Aqueous extract of *P. oleracea* L. seeds	*In vivo, * albino rats	10 mL/kg, 3 weeks, p.o.	↓ Epileptic hyper-excitability, GFAP, and lipid peroxidation	(84)
Hypoxia				
*P. oleracea* L. extracts	*In vivo, * male BALB/c mice	3 g/ml for 2 ml, twice a day for five days, p.o.	↑ Erythropoietin mRNA and protein expression in the cortex, activities of pyruvate kinase, phosphofructokinase, and LDH	(78)
Ethanol extract of *P.* *oleracea* L.	*In vivo, * ICR mice	100, 200, 400 mg/kg, 7 days, p.o.	↑ Survival time in normobaric and chemical hypoxia models, glycolysis enzymes, and ATP levels	(25)
Ethanol extract of *P.* *oleracea* L.	*In vivo, * ICR mice	0.5, 1, or 2 mg/kg, 7 days, p.o.	- Stabilized HIF-1α ↑ Neuron viability, endogenous erythropoietin expression at both mRNA and protein levels ↓ Serum neuron-specific enolase level, activity of caspase-3 in neuron, pathological damages	(89)
*P. oleracea* L. seed oil	*In vivo, * rats	0.25, 0.50, and 0.75 mg/kg, 30 days, p.o.	↓ Total volume of brain stroke, median score of neurological violations	**(** 97 **)**

ACTH: adrenocorticotropic hormone; ATP: adenosine triphosphate; GFAP: glial fibrillary acidic protein; HIF-1α: hypoxia-inducible factor-1α; IP: intraperitoneal; LDH: lactate dehydrogenase; MDA: malondialdehyde; mRNA: messenger ribonucleic acid; PO: oral

**Table 4:** Effect of *Purtulaca oleracea* L. on pain

**Compound **	**Study design**	**Doses/Duration**	**Results**	**References**
*P. oleracea* L. seed hydro-alcoholic extract	*In vivo, * male BALB/c mice	12.5, 50, and 100 mg/kg, IP	↑ Pain threshold	(103)
*P. oleracea * L. leaf and seeds methanol extract	*In vivo, * Swiss albino mice	300 and 500 mg/kg, p.o.	-Reducing inflammation: seed extract was more efficient than the leaf extract -Analgesic: leaf extract was more effective than seed extract in the early phase, while seed extract was more officious in the late phase	(104)
*P. oleracea * L. hydro-alcoholic extract	*In vivo, * Wistar rats	100 and 200 mg/kg, 14 days, IP	↓ Pain-related behaviors, oxidative damage (MDA), inflammatory cytokines including TNF-α and IL-1β in the spinal cord	(105)
*P. oleracea * L. cream (2% concentrated purslane alcoholic extract with cold cream)	Clinical trial, nursing mothers	2% purslane cream (right after nursing on the nipple skin as much as a knuckle, three times a day), 8 days	**↓ ** Pain intensity	(106)
*P. oleracea * L. ointment (5% concentrated purslane alcoholic extract and 95% ointment base)	Clinical trial, elderly patients with chronic musculoskeletal pain	5% purslane cream (on the knee and back joints at night before sleeping, for 8 hr), 2 weeks	**↓ **Sensory and emotional dimensions of pain, the severity of pain	(107)

IL-1β: interleukin-1 beta; IP: intraperitoneal; MDA: malondialdehyde; PO: oral; TNF-α: tumor necrosis factor-alpha

## Conclusion

Purslane is effective against a variety of nervous system disorders, and thus it could be a promising therapeutic compound for managing and treating some nervous system disorders, including Alzheimer’s disease, Parkinson’s disease, depression, epilepsy, hypoxia, and pain through multiple mechanisms such as antioxidant, anti-inflammatory, and anti-apoptotic effects, though finding more precise mechanisms of purslane neuroprotective and neuroregenerative effects require additional research. 

It is also worth mentioning that studies on herbal medications should be taken into account more because many herbal medicines’ safety and efficacy are still unknown. In addition, more reliable trials are needed to assess the safety and efficacy of various purslane components in treating different nervous system illnesses. Furthermore, the possible antagonistic and synergistic effects of multi-component purslane mixtures should be investigated using a combination of physiological, pharmacological, bioavailability-centered, and pharmacokinetic techniques. Traditional purslane formulations should not be used for lengthy periods or at large doses until more comprehensive toxicity studies are available. The new findings could help expand the medicinal values of purslane in the future, as well as its application in modern medicine.

## Authors’ Contributions

JJ rose the notion and helped in gathering the data. MGR wrote the manuscript. Both authors approved the final version of the manuscript.

## Conflicts of Interest

The authors declare that they have no conflicts of interest.
